# Demonstrating the reduction of enteric viruses by drinking water treatment during snowmelt episodes in urban areas

**DOI:** 10.1016/j.wroa.2021.100091

**Published:** 2021-01-25

**Authors:** Émile Sylvestre, Michèle Prévost, Jean-Baptiste Burnet, Xiaoli Pang, Yuanyuan Qiu, Patrick Smeets, Gertjan Medema, Mounia Hachad, Sarah Dorner

**Affiliations:** a NSERC Industrial Chair on Drinking Water, Department of Civil, Geological, and Mining Engineering, Polytechnique Montreal, Montreal, Quebec, H3C 3A7, Canada; b Canada Research Chair in Source Water Protection, Department of Civil, Geological, and Mining Engineering, Polytechnique Montreal, Montreal, Quebec, H3C 3A7, Canada; c Department of Laboratory Medicine and Pathology, University of Alberta, 116th & 85 Ave, Edmonton, AB, T6G 2R3, Canada; d Public Health Laboratory, Alberta Precision Laboratories, 8440-112nd, Edmonton, AB, T6G 2J2, Canada; e KWR Water Research Institute, Groningenhaven 7, 3433 PE, Nieuwegein, the Netherlands; f Sanitary Engineering, Department of Water Management, Faculty of Civil Engineering and Geosciences, Delft University of Technology, P.O. Box 5048, 2600GA, Delft, the Netherlands

**Keywords:** Drinking water, Enteric viruses, β-d-glucuronidase, Risk assessment

## Abstract

This study investigates short-term fluctuations in virus concentrations in source water and their removal by full-scale drinking water treatment processes under different source water conditions. Transient peaks in raw water faecal contamination were identified using *in situ* online β-d-glucuronidase activity monitoring at two urban drinking water treatment plants. During these peaks, sequential grab samples were collected at the source and throughout the treatment train to evaluate concentrations of rotavirus, adenovirus, norovirus, enterovirus, JC virus, reovirus, astrovirus and sapovirus by reverse transcription and real-time quantitative PCR. Virus infectivity was assessed through viral culture by measurement of cytopathic effect and integrated cell culture qPCR. Virus concentrations increased by approximately 0.5-log during two snowmelt/rainfall episodes and approximately 1.0-log following a planned wastewater discharge upstream of the drinking water intake and during a β-d-glucuronidase activity peak in dry weather conditions. Increases in the removal of adenovirus and rotavirus by coagulation/flocculation processes were observed during peak virus concentrations in source water, suggesting that these processes do not operate under steady-state conditions but dynamic conditions in response to source water conditions. Rotavirus and enterovirus detected in raw and treated water samples were predominantly negative in viral culture. At one site, infectious adenoviruses were detected in raw water and water treated by a combination of ballasted clarification, ozonation, GAC filtration, and UV disinfection operated at a dose of 40 mJ cm^−2^. The proposed sampling strategy can inform the understanding of the dynamics associated with virus concentrations at drinking water treatment plants susceptible to de facto wastewater reuse.

## Highlights

• β-d-glucuronidase guided monitoring was used to investigate virus reduction.
• Raw water virus concentrations increased by 1.0-log during β-d-glucuronidase peaks.
• Virus removal during snowmelt events was similar or better than in baseline conditions.
• Infectious adenoviruses were detected after UV disinfection.

## Graphical abstract

**Figure:**
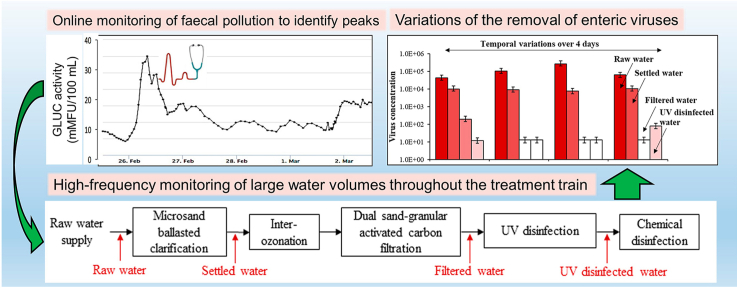

## 1 Introduction

Accurate data on the physical removal and inactivation of enteric viruses by engineered water treatment processes is essential to the implementation of risk-based preventive approaches to ensure drinking water safety (WHO, 2017). Virus removal performances are commonly assessed by spiking cultured or isolated virus stocks. These performances have been estimated at bench- and pilot plant-scales by plaque assays (Hijnen and Medema, 2010) or quantitative PCR (qPCR) assays (Kato et al., 2018; Shirasaki et al., 2017). A limited number of studies also investigated the removal of viruses under full-scale operating conditions in drinking water treatment plants (DWTPs) (Albinana-Gimenez et al., 2009; Teunis et al., 2009). However, these removal performances are usually measured under random raw water quality conditions, and little is known about specific removal performances during hydrometeorological events.

Coagulation and flocculation are critical steps defining the efficacy of settling and chemically assisted filtration, especially during intermittent changes in raw water quality. Inadequate floc formation, floc breakdown, and filter overloading can lead to increased amounts of particles in finished water, which can render virus disinfection ineffective (Hejkal et al., 1979). Natural organic matter (NOM) concentration in raw water can increase following rainfall events (Hurst et al., 2004), which can interfere with virus flocculation performance (Nasser et al., 1995). Furthermore, coagulation with hydrolyzing metal salts can perform less well at low water temperature due to lower solubility and slower kinetics of the metal hydroxides (Driscoll and Letterman, 1988; Kang and Cleasby, 1995) and poor floc formation (Hanson and Cleasby, 1990; Morris and Knocke, 1984). Snowmelt episodes associated with high virus concentrations in river water during cold months could, thus, represent periods of higher viral risks for drinking water consumers (Sokolova et al., 2015). However, the identification and characterization of peaks in raw water viral contamination at drinking water intakes remain challenging (Westrell et al., 2006).

The automatization of rapid methods for detecting indicators of faecal contamination in surface water (Koschelnik et al., 2015; Ryzinska-Paier et al., 2014) is stimulating the development of new strategies to characterize short-term variations in raw water contamination at drinking water intakes. Online near real-time monitoring of β-d-glucuronidase (GLUC) activity has recently been used to measure the frequency, duration and amplitude of faecal contamination peaks at an urban DWTP (Burnet et al., 2019b). Furthermore, event-based monitoring strategies triggered by GLUC activity were recently developed to characterize *Escherichia coli* (*E. coli*) peaks (Sylvestre et al., 2020a) and protozoan pathogens peaks (Sylvestre et al., 2020b) at DWTPs. However, it is unclear whether online GLUC activity can be used to identify peaks in source water viral contamination. This knowledge gap limits the application of online GLUC activity monitoring for water safety management and warrants further investigations (Demeter et al., 2020).

Therefore, the main objective of this work is to quantitatively describe short-term fluctuations in virus concentrations in source water and their removal by full-scale drinking water treatment processes under different source water conditions: baseline (background) conditions and peak conditions during snowmelt periods. Online β-d-glucuronidase (GLUC) activity measurements and local meteorological conditions were considered to inform sampling strategies. Large volumes of raw water (50–2200 L) and treated water throughout the treatment train were concentrated to quantify multiple enteric viruses, including norovirus, rotavirus, reovirus, sapovirus, astrovirus, enterovirus, adenovirus, and a non-enteric virus John Cunningham (JC) virus by reverse transcription (RT) and real-time quantitative PCR (qPCR). Virus infectivity of cultivable viruses was also assessed using the cytopathic effect in cell culture and integrated cell culture with qPCR (ICC-qPCR).

## 2 Materials and methods

### 2.1 Catchment description

Sampling campaigns were carried out at two drinking water treatment plants (DWTPs) treating water from the Milles Iles River in the greater Montreal area in Quebec, Canada. The river has a length of 40 km, an average water discharge of 286 m^3^ s^−1^. It is one of the major rivers of the Montreal Archipelago, where the Ottawa River meets the Saint Lawrence River. Drinking water intakes A and B are located at the middle point and the end of the river, respectively. Locally, this river and its tributaries are under the direct influence of a series of small watersheds totalling 1190 km^2^. There are 184 combined sewer overflows (CSOs) outfalls and 14 municipal wastewater treatment plants (WWTPs) outfalls located in these small watersheds. CSOs discharges can occur intermittently during rainfall or heavy snowmelt events. Most of these WWTPs are using aerated ponds or combined biological and physicochemical treatment processes. Diffuse pollution sources may also contribute to viral contamination of animal origin in drinking water supplies because river tributaries are draining agricultural lands. Spring snowmelt freshet usually occurs between February and April in Southern Quebec, and it is the critical period for microbial peaks at drinking water intakes located in this river (Burnet et al., 2019b).

### 2.2 Drinking water treatment description

**Fig. 1:**
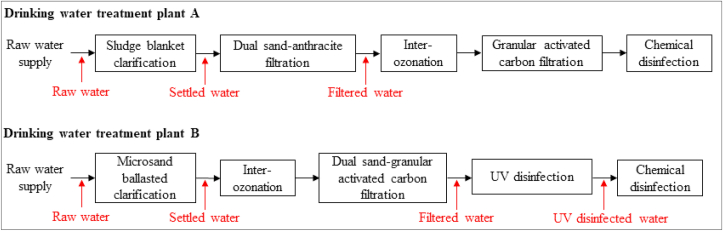
Unit processes involved in the treatment train of drinking water treatment plants A and B and sampling points (red). (For interpretation of the references to colour in this figure legend, the reader is referred to the Web version of this article.)

An overview of unit processes involved in the treatment train of each DWTP and the location of sampling points is illustrated in Fig. 1. For both DWTPs, Supervisory Control and Data Acquisition (SCADA) data (flow rate, turbidity, pH, ammonia, coagulant dosage, disinfectant residual) were collected to relate these parameters to the observed removal of viruses.

During the sampling campaigns, DWTP A was operated at a capacity of 40,000 m^3^ d^−1^, approximately 35% of the design rate (110,000 m^3^ d^−1^). The raw water was coagulated with aluminum sulfate “alum” (Al_2_(SO_4_)_3•_18 H_2_O; dosing rate: 50 mg L^−1^) and silica sand (SiO_2_; dosing rate: 2 mg L^−1^) at pH 6.0 and processed by a floc blanket clarifier. A first-stage dual sand-anthracite filtration then processed the settled water (10 m h^−1^; 30 cm sand-bottom and 60 cm anthracite-top). The filtered water then passed through inter-ozonation (dose rate: 1.2 mg L^−1^ O_3_), second-stage granular activated carbon (GAC) filtration (5–10 m h^−1^; 200 cm of activated carbon), and chemical disinfection with chlorine dioxide (2.3 mg L^−1^ ClO_2_).

DWTP B was operated at a capacity of 46,800 m^3^ d^−1^, approximately 40% of the design rate (120,000 m^3^ d^−1^). The raw water was processed by an ACTIFLO® microsand ballasted clarifier (Veolia Water Technologies, QC, Canada). During the sampling period, alum (Al_2_(SO_4_)_3_; dosing rate:15 mg L^−1^), polyaluminosilicate-sulfate (PASS-10; dosing rate: 50 mg L^−1^), cationic polyacrylamide (CPAM; dosing rate: 0.25 mg L^−1^) and silica sand (SiO_2_; dosing rate: 4 g L^−1^) were added in 1 °C raw water at pH 6.7. The settled water then passed through inter-ozonation (dosing rate:1.0 mg L^−1^ O_3_; Ct_10_: 0.6 mg L^−1^ min^−1^) for 20–22 min and is processed by dual sand and granular activated carbon (GAC) filters (10 m h^−1^, 15 cm sand-bottom and 140 cm activated carbon-top). The filtered water then went through low pressure (LP, λ = 254 nm) UV disinfection (reduction equivalent dose: 40 mJ cm^−2^, Wedeco BX 3200; Xylem Water Solutions, Herford, Germany) and chemical disinfection with sodium hypochlorite (dosing rate: 2.1 mg L^−1^ NaOCl).

### 2.3 Sampling strategy

Baseline (background) conditions and peak conditions during snowmelt periods were identified using β-d-glucuronidase (GLUC) activity measurements in raw water and local meteorological conditions (temperature, precipitation). GLUC activity was used as an indicator of the faecal contamination level. An automated rapid monitoring system (ColiMinder™ VWMS GmbH, Vienna, Austria) was installed at each DWTP in February (1–2 months before significant snowmelt episodes) to monitor variations of GLUC activity in raw water. GLUC activity was measured every 1–3 h and was reported online in modified Fishman units (mMFU 100 mL^−1^). Turbidity levels were measured continuously in raw water and every 4 h in settled water, individual filter effluents, and combined effluent from all filters.

At DWTP A, the event-based sampling strategy was based on meteorological conditions (daily rainfall > 20 mm or air temperature > 5 °C over 24 h) and GLUC activity levels (variation > +5 mMFU 100 mL^−1^ over 1 h). Two events (Event A1 in February and Event A2 in April) were captured with this sampling strategy. Sequential grab raw water samples (110–500 L) were collected at a frequency of 4–6 h for around 24 h to obtain a virus concentration profile over time. Sequential grab samples of settled and filtered waters (300–600 L) were collected to match theoretical mean hydraulic residence times through clarification (3 h) and filtration (2 h) (C. Durivage, *personal communication*). Samples were not collected under baseline conditions.

At DWTP B, four sampling campaigns were carried out under peak conditions (2) and baseline conditions (2). On February 7, 2018, a planned discharge of raw sewage (4 h) was undertaken to maintain the main sewer system at a municipal wastewater treatment plant (WWTP) located 5 km upstream of DWTP B. The WWTP serves a population of 37,000 residents and treats, on average, 28,000 m^3^ of raw sewage per day. In normal conditions, the wastewater is treated using aerated lagoons. The impact of this discharge on raw water quality at DWTP B was evaluated using GLUC activity to trigger grab sampling of raw water (Event B1). In March 2018, a rapid increase in GLUC activity (+20 mMFU/100 mL) was observed in dry weather conditions. Results from event-based sampling campaigns at DWTP A in 2017 showed that such variations in GLUC activity could indicate short-term variations >0.5-log in virus concentrations. We thus assumed that this period of high GLUC activity represented peak conditions in source water microbial concentrations (Event B2), even if local meteorological conditions did not suggest a significant impact of rainfall or rapid snowmelt on source water quality. Sequential grab samples of raw (50–200 L), settled (350–520 L), filtered (1000–2000 L), and UV disinfected (1200–2700 L) waters were collected over four days during Event B2. Baseline conditions were defined as periods of low GLUC activity level (<20 mMFU/100 mL) and dry weather conditions (daily rainfall < 1 mm and air temperature < 5 °C over 24 h). Grab samples of settled, filtered, and UV disinfected waters were collected under baseline conditions. Theoretical mean hydraulic residence times throughout clarification (1.5 h), filtration, and UV disinfection (1.5 h) were matched for each raw water sample (M. Marchand, *personal communication*).

### 2.4 Virus concentration method

An adsorption-elution method was applied to concentrate viruses from water samples using electropositive filters NanoCeram VS2.5-5 (Argonide Corp, Sanford, FL, USA). Samples were filtered on-site at the DWTPs under a constant flow rate of 5–15 L min^−1^. Pre-filters were not used in this study. A decontamination protocol was applied to prevent cross-contamination during the repeated use of the filtration system. Before each use, the intake and cartridge housing modules were sterilized with 6% NaOCl for 30 min, rinsed with sterile ddH_2_O, and then dechlorinated with a sodium thiosulfate solution. After filtration, cartridges were stored and kept cool (between 1 and 10 °C) in a transport cooler and shipped to the University Alberta Hospital in Edmonton, AB, Canada, for processing within 48 h after the start of the field sample collection. Eight samples collected at DWTP B on February 28, 2018, could only be processed 96 h after the collection due to shipment delay. The elution and flocculation steps after filtration were performed to concentrate the viruses in the samples as previously described (Pang et al., 2012). In brief, viruses retained by the positively charged filter were eluted with 1 L of 1.5% beef extract (BE) buffer (pH 9.75). The eluate was further flocculated with FeCl_3_ and pH adjustment to 3.5 followed by centrifugation. The water concentrate was suspended in glycine buffer (0.5 mol/L glycine, pH 9.0) with a final volume of 15 mL. The pH of the suspension was adjusted to 7.2 ± 0.2. The concentrate was stored at −70 °C until assayed.

### 2.5 Nucleic acid extraction and quantification of enteric viruses by qPCR

Total nucleic acids were extracted from 200 μL of concentrated water samples and eluted in 50 μL RNase-free water using the MagaZorb® total RNA Prep kit (Promega, WI, USA). Nucleic acid extracts were tested for norovirus genogroup I and GII (GI/GII), rotavirus, sapovirus, astrovirus, generic adenovirus, enterovirus, JC polyomavirus, and reovirus. Quantification of viruses was performed by a two-step reaction (RT and qPCR) with the ABI PRISM 7500 Sequence Detection System (ABI) as previously described (Qiu et al., 2015, 2016). The primer and probes used for qPCR were published previously (Pang et al., 2012; Qiu et al., 2015, 2018). RT and qPCR were carried out as described previously (Pang et al., 2012). Salmon DNA was included as an internal control to monitor inhibition. An external standard curve was established for quantification of all eight viruses using the 875 bp DNA fragment of norovirus GII by 10-fold dilution from 10 to 1×10^6^ genome-copies (Qiu et al., 2016). Optimization of the panel qPCR assay for the eight viruses was performed by adjusting thermal cycler conditions and concentration of primers and probes to achieve similar qPCR efficiencies. Based on the standard curves and the cycle threshold values, the virus concentration (free or encapsidated genomes) was expressed as genome-copies per litre. Sample-specific recovery rates were not measured, but the recovery rates of qPCR-based assays were described in a previous study (Pang et al., 2012). The limit of detection (LOD) of qPCR-based assays was one genome copy per PCR reaction, which was equal to 2–140 genome-copies per 100 mL based on each sample’s volume and their concentrate volume.

### 2.6 Virus cell culture

Viral replication in cultures was determined by monitoring cytopathic effects (CPE). Infectivity of rotavirus, enterovirus, adenovirus, and reovirus was assessed in each sample using Buffalo green monkey kidney cells (BGM) and African rhesus monkey kidney cells (MA104) grown separately on Eagle’s MEM medium (Sigma, ON, Canada) as previously described (Qiu et al., 2015). Integrated cell culture (ICC)-qPCR assay was used to evaluate the presence of infectious virions in the sample, as detailed by Qiu et al. (2015).

### 2.7 Quantification of virus concentrations

A hierarchical Bayesian framework was adopted to evaluate virus concentrations. Two levels of analysis were specified to describe uncertainties related to the random error in sample collection and the analytical recovery due to losses during sample processing. Purified virus nucleic acids in each PCR $\left(N_{p}\right)$ were assumed to be Poisson-distributed with a mean $\lambda_{p}$ (Varughese et al., 2018). This model assumes that virus nucleic acids are fully disaggregated and randomly distributed in the water (i.e., homogeneous concentration) within the time and space from which the sample was collected. The virus to genome-copy ratio was assumed to be 1:1. The expected analytical recovery of the detection method was assumed to vary randomly among samples according to a beta distribution (Wu et al., 2014). The model can be written as:

(1) $$N_{p}\left[i\right]∼\text{Poisson}\left(\lambda_{p}\left[i\right]\right)$$

(2) $$\lambda_{p}\left[i\right]=\left[v\left[i\right]*\;V_{S}\left[i\right]*\frac{\;V_{PCR}\left[i\right]}{V_{P}\left[i\right]}\;\right]*r\left[i\right]$$

(3) r[i]∼Beta(α,β)

Where, v is the virus concentration, $V_{S}$ is the volume of raw of treated water filtered with the NanoCeram ® filter, $V_{PCR}$ is the volume for the PCR reaction, $V_{P}$ is the volume of the pellet (i.e., concentrated sample for the nucleic acid extraction), and r is the expected analytical recovery. Shape parameters (α,β) of the Beta distribution were estimated from recovery rates previously published for adenovirus 41 (n = 3; mean = 0.18, standard deviation (STD) = 0.03) and norovirus GII.4 (n = 3; mean = 0.19, STD = 0.03) spiked and concentrated from 10 L of river water by NanoCeram® filtration and assayed by real-time quantitative RT-PCR and PCR (Pang et al., 2012). Beta distributions for adenovirus 41 and norovirus GII.4 were used to describe the recovery rates of other DNA and RNA viruses.

The Bayesian analysis was conducted in R (v3.4.1) via rjags (v4-6) (Plummer, 2013). The uncertainty in parameter values was evaluated using a Markov Chain Monte Carlo procedure. Four Markov chains were run for 10^4^ iterations after a burn-in phase of 10^3^ iterations. The Brooks-Gelman-Rubin scale reduction factor was considered to monitor the convergence of the four chains (Gelman and Shirley, 2011). A conjugate gamma prior was selected to describe the virus concentration v of the Poisson model. The shape and rate parameters of the gamma prior were both set to 0.001. This prior reflects practically no prior knowledge. The posterior mean of the estimate virus concentration and its 95% credibility interval were reported. The R code used to quantify virus concentrations is provided in the supplementary material.

### 2.8 Quantification of virus removal

Treatment removal performances were quantified using an empirical approach. Point estimates of the log-removal (LR) across a treatment unit (paired sample) were calculated as follows:

(4) $$\text{LR}={\log\;}_{10}\left(\frac{C_{in}}{C_{out}}\right)$$

where $C_{in}$ and $C_{out}$ are the best estimate virus concentration per sample (genome-copies L^−1^) before and after treatment, respectively. The uncertainty in virus concentrations was not considered in the quantification of LR. The limit of detection was considered in the calculation when $C_{out}$ was not quantified. When multiple grab samples were collected in sequence over an event, the mean (effective) log-removal (${\text{LR}}_{\text{effective}}$) was calculated as follows:

(5) $${\text{LR}}_{\text{effective}}={\log\;}_{10}\left(\frac{{\bar{C}}_{in}}{{\bar{C}}_{out}}\right)$$

Each treatment step of the DWTP was assumed to behave as a plug flow reactor operated hydraulically at a steady state. The theoretical hydraulic residence time was assumed to be a valid approximation of the retention time of the viruses.

## 3 Results

### 3.1 Short-term fluctuations in virus concentrations in source water

**Fig. 2:**
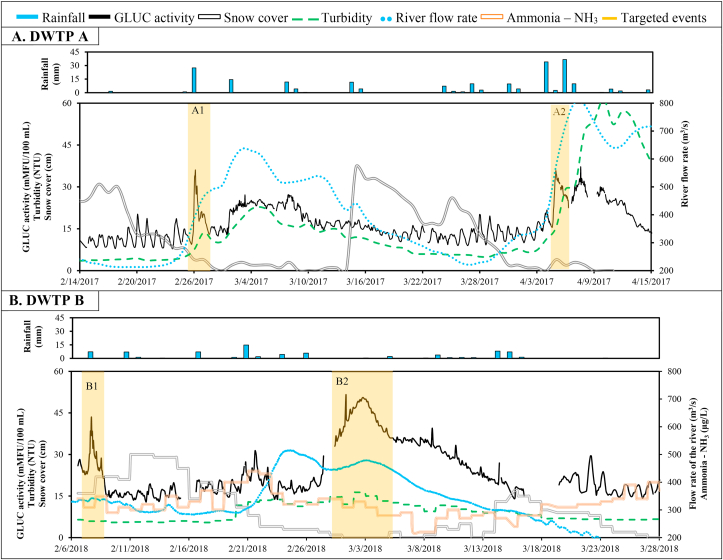
Time series of daily rainfall, β-d-glucuronidase (GLUC) activity, snow cover, raw water turbidity, raw water ammonia concentration and river flow rate during snowmelt periods in 2017 and 2018 at drinking water treatment plants (DWTPs) A and B. Yellow rectangles indicate targeted events. (For interpretation of the references to colour in this figure legend, the reader is referred to the Web version of this article.)

Short- and long-term time series of GLUC activity, turbidity, ammonia, and hydrometeorological variables at drinking water intakes A and B are presented in Fig. 2 and Supplementary Fig. 1. At DWTP A, the sampling strategy was triggered by local meteorological conditions and short-term GLUC activity variations. The turbidity level and the river flow rate peaked several days after the GLUC activity for the two targeted events (Supplementary Fig. 1).

**Fig. 3:**
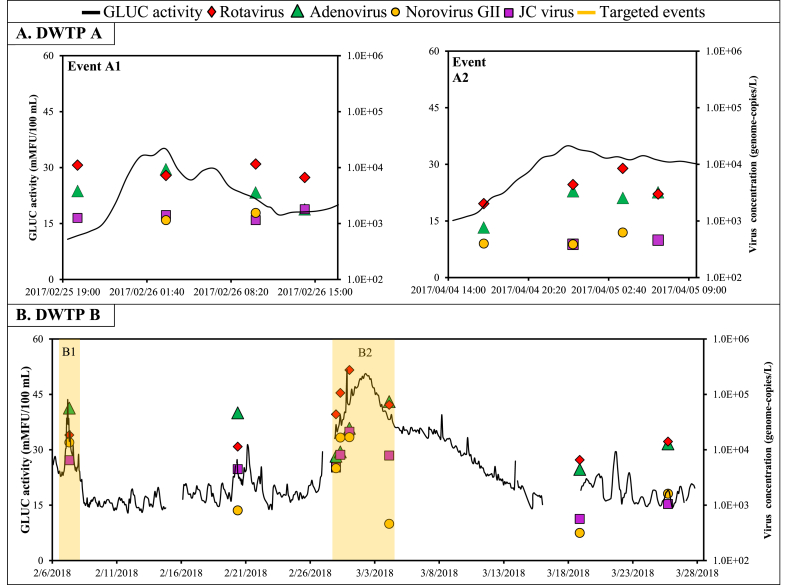
Time series of β-d-glucuronidase (GLUC) activity and rotavirus, adenovirus, norovirus GII, and JC virus concentrations during snowmelt episodes at drinking water treatment plants (DWTPs) A and B. Yellow rectangles indicate targeted events. (For interpretation of the references to colour in this figure legend, the reader is referred to the Web version of this article.)

**Fig. 4:**
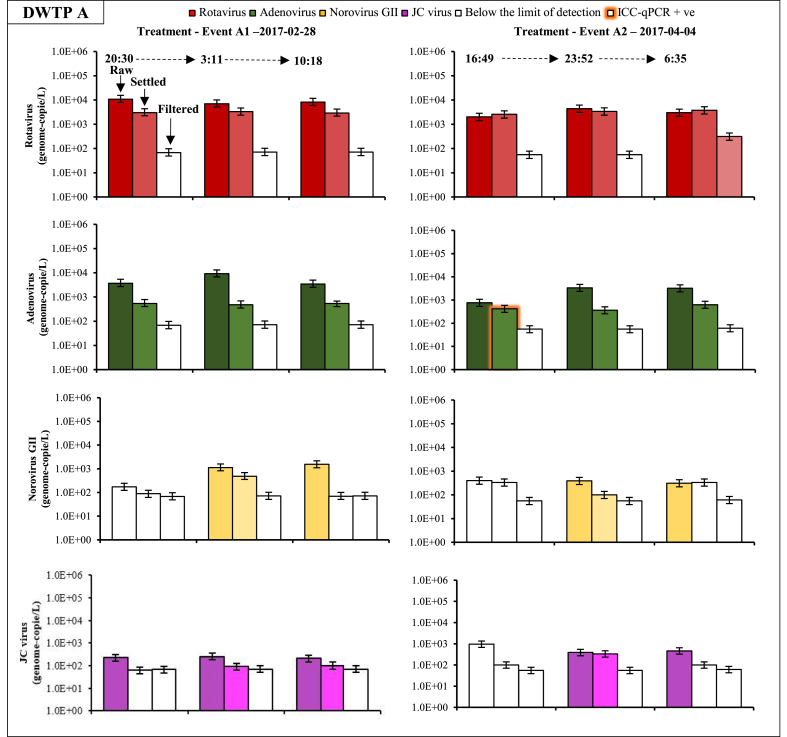
Histograms for rotavirus, adenovirus, norovirus, and JC virus concentrations in raw water, settled water, and filtered water during events A1 and A2 at drinking water treatment plant (DWTP) A. Error bars represent the 95% credibility interval on the virus concentration assuming that purified virus nucleic acids in each PCR are Poisson-distributed. Columns with no colour represent the limit of detection. Orange glowing bars represent samples positive by ICC-qPCR. (For interpretation of the references to colour in this figure legend, the reader is referred to the Web version of this article.)

Rotavirus, adenovirus, norovirus GII, and JC virus were detected in most samples during the two snowmelt events (Table 1). Raw water concentrations peaked at around 10^4^ genome-copies L^−1^ for adenovirus and rotavirus and 10^3^ genome-copies L^−1^ for norovirus GII and JC virus (Fig. 3A). Rotavirus and adenovirus concentrations increased by approximately 0.5- and 1.0-log, respectively, during these 24-h periods. The uncertainties related to the random error in sample collection and the analytical error are shown with credible intervals in Fig. 4. These 95% credibility intervals indicate that the uncertainty on the virus concentration per sample is approximately 0.5-log.

**Table 1:** Number of positive samples by qPCR for each virus at each treatment step at drinking water treatment plants (DWTPs) A and B.

n	DWTP A			DWTP B			
	Raw water	Floc blanket clarif.	Rapid sand filtration	Raw water	Ballasted clarif.	Ozonation + GAC filtration	UV disinf.
	8 (%)	6 (%)	6 (%)	8 (%)	6 (%)	6 (%)	6 (%)
Rotavirus	8 (100)	6 (100)	1(17)	8 (100)	6 (100)	3 (50)	3 (50)
Adenovirus	8 (100)	6 (100)	0	8 (100)	6 (100)	2 (33)	0
Norovirus GI	1 (13)	0 (0)	0	8 (100)	0	0	0
Norovirus GII	6 (75)	3 (50)	0	8 (100)	0	0	0
JC virus	6 (75)	4 (66)	0	8 (100)	3 (50)	0	0
Enterovirus	0	0	0	3 (38)	0	0	0
Reovirus	0	0	0	0	0	0	0
Astrovirus	0	0	0	2 (25)	0	0	0
Sapovirus	1 (13)	0	0	1 (13)	0	0	0

At DWTP B, the sampling strategy to capture peak events in raw water viral contamination was based on increases in GLUC activity only. Local meteorological conditions were not considered to trigger sampling during peak conditions. On February 7, raw water GLUC activity increased from 20 to 40 mMFU 100 mL^−1^ about 10 h following the planned wastewater discharge (Event B1). This peak had a duration of approximately 24 h (Fig. 2B). In March, the raw water GLUC activity gradually increased from 20 to 50 mMFU 100 mL^−1^ over five days without significant cumulative rainfall (<10 mm) and cumulative snowmelt (<10 cm) over the ten days preceding the GLUC activity peak (Event B2). Baseline conditions (GLUC activity level <20 mMFU/100 mL and dry weather conditions) were assessed approximately two weeks after Event B2. Turbidity and ammonia levels did not peak following the planned wastewater discharge and during the GLUC activity peak in dry weather conditions (Supplementary Fig. 1).

Concentrations of rotavirus, adenovirus, norovirus GII, and JC virus were about 1.0-log higher during peak event conditions than during baseline conditions (Fig. 3B). Enterovirus, astrovirus and sapovirus were sporadically measured in event-based samples. Raw water concentrations peaked at around 10^5^ genome-copies L^−1^ for adenovirus and rotavirus and 10^4^ genome-copies L^−1^ for norovirus GII and JC virus. These concentrations were approximately 1.0-log lower than treated wastewater effluent concentrations and approximately 2.0-log lower than raw sewage concentrations as measured at the upstream WWTP (Supplementary Fig. 1).

### 3.2 Removal by full-scale treatment processes using qPCR data

**Fig. 5:**
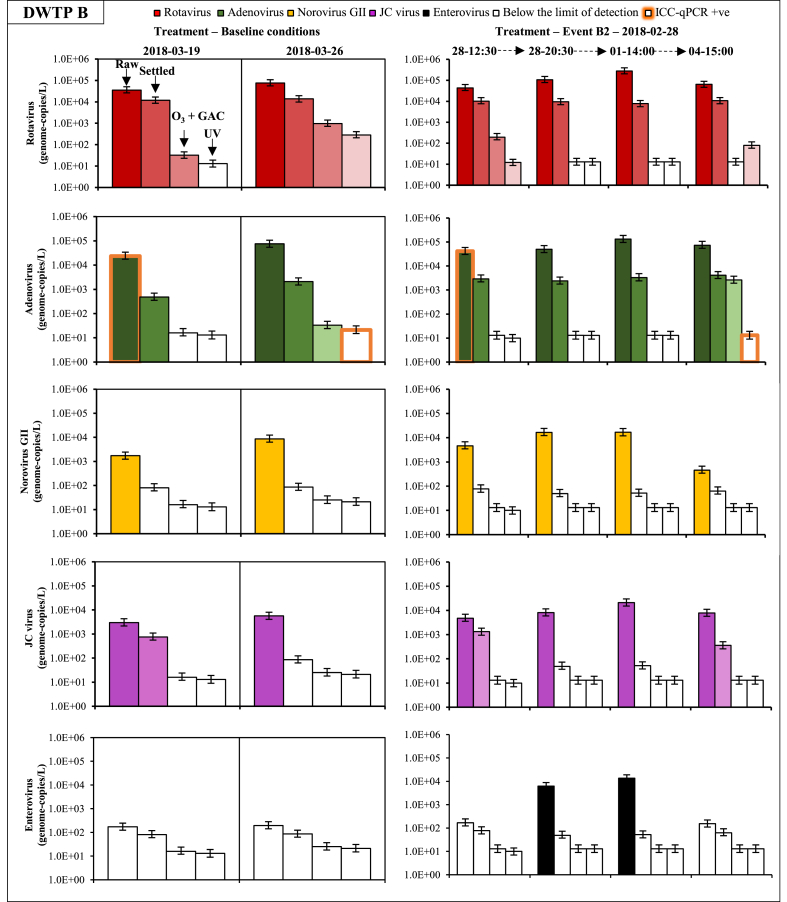
Histograms for rotavirus, adenovirus, norovirus GII, JC virus, and enterovirus concentrations in raw water, settled water, filtered water and UV disinfected water under baseline and event conditions (Event B2) at drinking water treatment plant (DWTP) B. Error bars represent the 95% credibility interval on the virus concentration assuming that purified virus nucleic acids in each PCR are Poisson-distributed. Columns with no colour represent the limit of detection. Orange glowing bars represent sample positives by ICC-qPCR. (For interpretation of the references to colour in this figure legend, the reader is referred to the Web version of this article.)

Time series of removal of viruses throughout the treatment trains are presented in Figs. 4 and 5. Error bars represent the 95% credibility interval on the concentration due to the analytical error and the random distribution of the genome-copies in the sample. At both DWTPs, the concentration of 300–520 L of settled water allowed the quantification of rotavirus and adenovirus (>300 genome-copies/L) in all samples. Norovirus GII was sporadically detected in settled water at DWTP A but not at DWTP B. At both DWTPs, reovirus, sapovirus, astrovirus, and enteroviruses were not detected in any samples collected throughout the treatment train.

Variations in removal performance for rotavirus, adenovirus, norovirus GI and GII, JC virus during baseline and event conditions are presented in Supplementary Table 1. Raw and settled water turbidity levels during sample collection are shown in Supplementary Table 2. For adenovirus, log-removals varied from 0.3 to 1.3-log for floc blanket clarification (DWTP A) and from 1.2 to 1.7-log for ballasted clarification (DWTP B). For rotavirus and norovirus GII, log removals by floc blanket clarification were negligible (≤ 0.5-log). In contrast, log removals by ballasted clarification varied from 0.5 to 1.6-log for rotavirus and from >0.9 to >2.5-log for norovirus GII. Overall, enteric viruses were removed to a higher degree by ballasted clarification than floc blanket clarification.

**Fig. 6:**
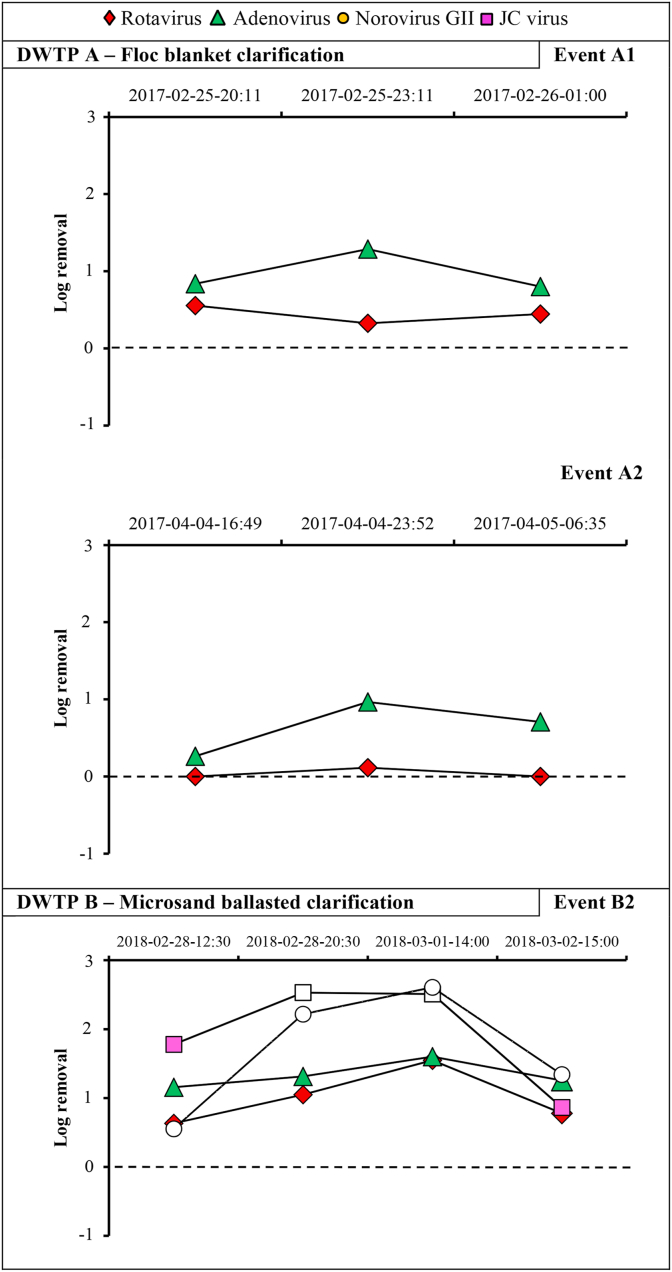
Change in virus removal performances of coagulation/flocculation in response to enteric virus peak concentrations in raw water during snowmelt episodes at drinking water treatment plants (DWTPs) A and B. White circles and squares represent minimum removal performance values due to the inability to quantify the virus in settled water (below the limit of detection).

Results from sequential grab samples show that the coagulation/flocculation of viruses did not deteriorate during these snowmelt episodes (Fig. 6); peak concentrations of adenovirus were buffered by coagulation/flocculation at both DWTPs. A buffering effect was also observed for rotavirus and JC virus at DWTP B.

The concentration of 500 to 2200 L did not allow us to quantify the log-removal of enteric viruses by filtration accurately. However, virus concentrations were sporadically quantified at DWTP A after filtration (rotavirus) (Fig. 4) and at DWTP B after a combination of ozonation and GAC filtration (adenovirus and rotavirus) and UV disinfection (rotavirus) (Fig. 5).

### 3.3 Inactivation of viruses using cell culture

The evaluation of virus infectivity using ICC-qPCR shows that rotavirus and enterovirus were predominantly negative in viral culture in raw and treated water samples at DWTPs A and B (Table 2). Reovirus was mostly positive in raw water samples but not in treated water samples at DWTPs A and B. At DWTP B, infectious adenoviruses were detected in 50% of the raw water samples (4/8) and 33% of the UV disinfected water samples (2/6).

**Table 2:** Detection of infectious viruses in water samples by cell culture and integrated cell culture (ICC) qPCR.

	n	DWTP A			DWTP B			
		Raw water	Floc blanket clarif.	Rapid sand filtration	Raw water	Ballasted clarif.	Ozonation + GAC filtration	UV disinf.
		8	6	6	8	6	6	6
CPE	Positive viral culture (%)	8 (100)	3 (50)	2 (33)	8 (100)	1 (16)	0 (0)	0 (0)
ICC-qPCR (positive samples)	Rotavirus	0	0	0	0	0	0	0
	Adenovirus	0	1	0	4	0	0	2
	Enterovirus	1	0	0	0	0	0	0
	Reovirus	8	1	2	7	0	0	0
	Unknown^a^	0	1	0	0	1	0	0

a Unknown: Samples showed CPE in the cell culture but could not be identified for a specific virus by ICC-qPCR.

## 4 Discussion

### 4.1 Characterization of viral contamination peaks in source water

A monitoring strategy triggered by automated online GLUC activity measurements and local meteorological conditions was proposed to characterize peaks in raw water viral contamination and their removal by treatment processes. This monitoring strategy was designed to provide insights on the amplitude of viral contamination peaks during snowmelt episodes at DWTPs located in highly urbanized areas. The adequate characterization of these short-term fluctuations is important in microbial risk assessment as the long-term risk may be driven by the occurrence of pathogen concentration peaks (Smeets et al., 2010; Teunis et al., 2004). GLUC activity was selected to trigger virus sampling because recent technological development enables its automated near-real-time measurement (Koschelnik et al., 2015). Furthermore, a high-resolution GLUC activity monitoring data set was available to study catchment dynamics at DWTP A (Burnet et al., 2019b).

At DWTP A, this sampling strategy allowed the detection of increases of approximately 0.5-log in adenovirus and rotavirus concentrations in raw water following two snowmelt/rainfall events. Short-term variations of approximatively 1.0-log in norovirus and adenovirus concentrations were previously reported in wet weather conditions in river water influenced by wastewater discharges (Hata et al., 2014). At DWTP B, GLUC activity monitoring allowed measuring increases in virus concentrations of approximately 1.0-log following a planned wastewater discharge upstream of the drinking water intake and during a GLUC activity peak in dry weather conditions. Concentrations of viruses in raw water during peak conditions were approximately 1.0-log higher at DWTP B (downstream) than DWTP A (upstream). Although virus concentrations were not quantified during the same year at DWTP A (2017) and DWTP B (2018), findings from the current study suggest that virus concentrations increased along an urban river influenced by numerous wastewater discharges. Increases in detection frequency and virus concentrations along rivers were also reported for major urban centers in France (Prevost et al., 2015) and in Alberta, Canada (Pang et al., 2019). In our study, the monitoring of virus at a WWTP upstream DWTP B also indicated that peak concentrations in raw water were around 2.0-log lower than virus concentrations in raw sewage and around 1.0-log lower than virus concentrations in treated wastewater effluent (aerated lagoons).

GLUC activity and virus concentrations peaked at drinking water intakes following rainfall/snowmelt events and dry weather conditions. The latter short-term trends may reflect faecal contamination transported over long distances and interfering enzymatic activity by non-fecal compounds (Fiksdal and Tryland, 2008). It has been established that viable but non-culturable (VBNC) *E. coli* can contribute to the GLUC activity signal (Burnet et al., 2019a; Ender et al., 2017; Garcia-Armisen et al., 2005; Stadler et al., 2016), and that VBNC *E. coli* decreased much more slowly than culturable *E. coli* (Servais et al., 2009). In our study, virus concentrations were quantified using a qPCR method, which detects the DNA/RNA from viable and nonviable viruses. Qualitative results (presence or absence of infectious viruses) obtained with ICC-qPCR indicated that adenovirus, rotavirus, and enterovirus found in samples of source water were mostly not infectious. Detailed investigations confirming these findings in other areas and under different environmental conditions are needed to determine whether GLUC activity is a generally useful trigger for viral contamination sampling.

Online physicochemical measurements or other microbial indicators might be used instead of GLUC activity to trigger virus sampling. In this study, turbidity did not simultaneously peak with virus concentrations during captured events. Viral contamination during snowmelt- and rainfall-runoff events may be dominated by point sources (CSO and WWTP discharges), whereas turbidity may come from the whole watershed.

### 4.2 Virus-type specific removal by full-scale treatment processes

The concentration of large volumes (50–600 L) of raw and settled water allowed characterizing the full-scale removal performance of a floc blanket clarifier (DWTP A) and a ballasted clarifier (DWTP B) for rotavirus and adenovirus under different source water conditions. Other enteric viruses were only sporadically detected in settled water, suggesting that rotavirus and adenovirus should preferably be selected as reference viruses to quantify the removal of enteric viruses by full-scale treatment processes.

The removal performance of physical treatment processes (log-removal) is commonly assumed to be a first-order process with respect to the influent concentration of the virus (i.e., the same fraction of viruses is removed regardless of the influent concentration) (Haas and Trussell, 1998). However, short-term increases in the removal performances of coagulation/flocculation processes were observed during raw water peak events for adenovirus at DWTP A, and for adenovirus and rotavirus at DWTP B. These results suggest that coagulation/flocculation processes do not operate under steady-state conditions but dynamic conditions in response to source water conditions. According to the Smoluchowski theory of flocculation, variations in the influent particle concentrations may influence the aggregation rate of viruses during flocculation. While such an evaluation would be interesting, this study was not designed to investigate this hypothesis.

Even if large volumes of water (300–2700 L) were concentrated, the quantification of virus removal performances by chemically assisted filtration and disinfection remained a challenge because of detection limits. Rotavirus and adenovirus genomes were sporadically detected after filtration, ozonation, and UV disinfection, and infectious adenoviruses were detected after UV disinfection at DWTP B. Previous studies reported low removal of viruses and bacteriophages by GAC filtration at pilot plant scale (Guy et al., 1977; Hijnen et al., 2010) and high UV-resistance of adenovirus at a dose of 40 mJ cm^−2^ (Meng and Gerba, 1996; Thurston-Enriquez et al., 2003). However, a viral ozonation study demonstrated that a *Ct* value of 0.6 mg L^−1^ min^−1^ (calculated *Ct* value at DWTP B) should be sufficient to inactivate adenovirus type 40 by at least 4.0-logs in treated water (Thurston-Enriquez et al., 2005). The disparity between our results and those of Thurston-Enriquez et al. (2005) makes it difficult to conclude on the extent to which full-scale ozonation processes may inactivate naturally occurring adenovirus. Poor mixing and hydraulic conditions have been found to reduce the inactivation of *E. coli* by full-scale ozonation processes (Smeets et al., 2006). The hydraulics of the full-scale ozonation system assessed in our study may also limit the reduction of adenovirus. Site-specific source water data and full-scale removal data incorporated into quantitative microbial risk assessment (QMRA) could be used to evaluate drinking water treatment plant operation and investigate whether adenovirus is removed to an acceptably low level, especially if chlorine disinfection is not performed for maintaining a residual.

### 4.3 Limitations for the quantification of virus concentrations

Spatial heterogeneity and aggregation of viruses may have an impact on the quantification log-removal during full-scale treatment. In this study, viral genome-copies were assumed to be fully disaggregated (and thus randomly dispersed) in all samples. Overdispersion was not evaluated because sample replicates were not collected. Still, large-volume samples were used for concentrating the viruses in the samples (>300 L), which should minimize this source of uncertainty.

Owing to the limitations in analytical viral recovery data in the full-scale treatment, the uncertainty in method recovery performance was incorporated in virus concentration estimates using a beta distribution of recovery rates reported by Pang et al. (2012). The same laboratory recently reported slightly lower recovery rates (human adenovirus 2/4, n = 28; mean = 0.14, STD = 0.14; norovirus GII, n = 10, mean = 0.10, STD = 0.06) for wastewater samples subjected to secondary treatment (Li et al., 2019). Monitoring the efficiency of the virus concentration step with a process control could increase the accuracy of virus concentration estimates in raw water during hydrometeorological events (Hata et al., 2014). Recovery rates for samples collected after treatment processes may differ from those measured in raw water because of changes in matrix composition. However, Pang et al. (2012) did not observe significant differences in recovery rates among pure, tap and raw water samples for two RNA viruses (norovirus and echovirus) and one DNA virus (adenovirus 41). Nevertheless, the presence of alum and silica sand in settled water may influence analytical recovery efficiencies.

The presence of viral genomes after ozonation and UV disinfection is challenging to interpret because encapsidated genomes and free nucleic acids can be detected. Infectious rotaviruses in raw water were usually not detected by ICC-qPCR unless high concentrations of those viruses were present in raw water (>10^4^ genome-copies L^−1^). One of the reasons is that human rotavirus does not propagate efficiently in the continuous in vitro cell lines we used (MA104 and BGM) (Arnold et al., 2009; Ward et al., 1984). ICC-qPCR has limited value to assess the inactivation of viruses in water samples because this method only indicates the absence/presence of an infectious virus. Serial dilutions of wastewater and source water samples have recently been carried out to quantify human infectious virus concentrations by ICC-qPCR with the most probable number (MPN) method (Qiu et al., 2018; Schijven et al., 2019); however, the concentrations of naturally occurring viruses throughout full-scale drinking water treatment train may be too low for quantification using dilutions. Considering these limitations, the fact that positive infectious adenoviruses were found in treated water after a combination of advanced treatment processes point to the need to develop improved infectious virus detection methods.

## 5 Conclusions

Two full-scale drinking water treatment plants in Quebec, Canada, were selected to investigate short-term fluctuations in virus concentrations in source water and their removal by full-scale drinking water treatment processes under different source water conditions. The following conclusions are drawn:

• Event-based sampling targeted by online β-d-glucuronidase activity monitoring allowed detecting increases in adenovirus and rotavirus concentrations of approximately 0.5-log during two snowmelt/rainfall episodes and 1.0-log following a planned wastewater discharge upstream of the drinking water intake and during a β-d-glucuronidase activity peak in dry weather conditions.
• Detailed investigations in other urban areas and under different environmental conditions are needed to evaluate whether the β-d-glucuronidase activity could be a general useful trigger for viral contamination sampling.
• The concentration of large water volumes allowed characterizing the full-scale removal performance of coagulation/flocculation processes for rotavirus and adenovirus, but not for norovirus, enterovirus, and JC virus. These results indicate that rotavirus and adenovirus should preferably be selected as reference viruses to quantify the removal of enteric viruses by full-scale treatment processes.
• Increases in the removal of adenovirus by floc blanket clarification and of adenovirus and rotavirus by ballasted clarification were observed during peak virus concentrations in source water. These findings suggest that coagulation/flocculation processes do not operate under steady-state conditions but dynamic conditions in response to source water conditions.
• Limited effectiveness of UV disinfection against naturally occurring adenovirus was observed at current operative doses of 40 mJ cm^−2^ after a combination of ballasted clarification, ozonation, GAC filtration. This finding is consistent with results from previous inactivation studies under bench-scale conditions.
• More full-scale performance demonstrations are needed to quantitatively and reproducibly evaluate virus removal achieved by drinking water treatment plants that may be susceptible to de facto wastewater reuse.

## Declaration of competing interest

The authors declare that they have no known competing financial interests or personal relationships that could have appeared to influence the work reported in this paper.
