# Supplementary material for: Demonstrating the reduction of enteric viruses by drinking water treatment during snowmelt episodes in urban areas
Source: Water Res X. 2021 Jan 25;11:100091. doi: 10.1016/j.wroa.2021.100091 (PMC7868990; doi:10.1016/j.wroa.2021.100091)
Supplement: Multimedia component 1 [file mmc1.docx]

**Supplementary material**

**Journal:** Water Research

**Title:** Demonstrating the reduction of enteric viruses by drinking water treatment during snowmelt episodes in urban areas

**Authors:** Émile Sylvestre, Michèle Prévost, Jean-Baptiste Burnet, Xiaoli Pang, Yuanyuan Qiu, Patrick Smeets, Gertjan Medema, Mounia Hachad, Sarah Dorner

**Number of pages:** 6

**Number of Tables:** 2

Supplementary Table 1

Supplementary Table 2

**Number of Figures:** 1

Supplementary Figure 1

**Number of R codes:** 2

R code

**Supplementary Table 1.** Log-removal for rotavirus, adenovirus, noroviruses GI and GII, and JC virus via floc blanket clarification and rapid sand filtration at drinking water treatment plant (DWTP) A and via microsand ballasted clarification, biological activated carbon filtration, and UV disinfection at DWTP B. $\mathrm{LR}_{\mathrm{effective}}$ is the effective log-reduction during event conditions. The log-removal has a greater-than sign (>) when the removal was quantified using the limit of detection of the effluent sample.

| **DWTP A** | | |  | **DWTP B** | | | |
| --- | --- | --- | --- | --- | --- | --- | --- |
| **Sample id.** | **FBC^A^** | **RGF_sand_^B^** |  | **Sample id.** | **BC^C^** | **O_3_+RGF_GAC_^D^** | **UV** |
| **Rotavirus** | | |  | **Rotavirus** | | | |
| Event A1-01 | 0.5 | >1.6 |  | Baseline 1 | 0.5 | 2.6 | >0.4 |
| Event A1-02 | 0.3 | >1.7 |  | Baseline 2 | 0.7 | 1.1 | 0.5 |
| Event A1-03 | 0.4 | >1.6 |  | Event B2-01 | 0.6 | 1.7 | 0.6 |
| $LR_{eff.}$ Event A1 | 0.4 | 1.6 |  | Event B2-02 | 1.1 | >2.9 | - |
| Event A2-01 | 0.0 | >1.7 |  | Event B2-03 | 1.6 | >2.8 | - |
| Event A2-02 | 0.1 | >1.8 |  | Event B2-04 | 0.8 | 2.9 | 0.0 |
| Event A2-03 | 0.0 | 1.1 |  | $LR_{eff.}$ Event B2 | 1.1 | 2.2 | - |
| $LR_{eff.}$Event A2 | 0.0 | 1.4 |  | **Adenovirus** | | | |
| **Adenovirus** | | |  | Baseline 1 | 1.7 | >1.5 | - |
| Event A1-01 | 0.8 | >0.9 |  | Baseline 2 | 1.6 | 1.8 | >0.2 |
| Event A1-02 | 1.3 | >0.8 |  | Event B2-01 | 1.2 | >2.3 | - |
| Event A1-03 | 0.8 | >0.9 |  | Event B2-02 | 1.3 | >2.3 | - |
| $LR_{eff.}$ Event A1 | 1.0 | 0.9 |  | Event B2-03 | 1.6 | >2.4 | - |
| Event A2-01 | 0.3 | >0.9 |  | Event B2-04 | 1.2 | 0.2 | >2.3 |
| Event A2-02 | 1.0 | >0.8 |  | $LR_{eff.}$ Event B2 | 1.4 | 0.67 | - |
| Event A2-03 | 0.7 | >1.0 |  | **Norovirus GI** | | | |
| $LR_{eff.}$ Event A2 | 0.7 | 0.9 |  | Baseline 1 | >1.2 | - | - |
| **Norovirus GII** | | |  | Baseline 2 | >1.3 | - | - |
| Event A1-01 | - | - |  | Event B2-01 | >1.2 | - | - |
| Event A1-02 | 0.4 | >0.8 |  | Event B2-02 | >1.8 | - | - |
| Event A1-03 | >1.3 | - |  | Event B2-03 | >2.0 | - | - |
| $LR_{eff.}$ Event A1 | - | - |  | Event B2-04 | >1.1 | - | - |
| Event A2-01 | 0.1 | >0.8 |  | $LR_{eff.}$ Event B2 | 1.5 | - | - |
| Event A2-02 | >0.6 | - |  | **Norovirus GII** | | | |
| Event A2-03 | - | >0.7 |  | Baseline 1 | >1.3 | - | - |
| $LR_{eff.}$ Event A2 | - | - |  | Baseline 2 | >2.0 | - | - |
| **JC virus** | | |  | Event B2-01 | >1.8 | - | - |
| Event A1-01 | >0.6 | - |  | Event B2-02 | >2.5 | - | - |
| Event A1-02 | 0.5 | 0.1 |  | Event B2-03 | >2.5 | - | - |
| Event A1-03 | 0.3 | 0.2 |  | Event B2-04 | >0.9 | - | - |
| $LR_{eff.}$ Event A1 | 0.4 | - |  | $LR_{eff.}$ Event B2 | 2.2 | - | - |
| Event A2-01 | - | - |  | **JC virus** | | | |
| Event A2-02 | 0.1 | >0.8 |  | Baseline 1 | 0.6 | >1.6 | - |
| Event A2-03 | >0.7 | - |  | Baseline 2 | 1.8 | - | - |
| $LR_{eff.}$ Event A2 | - | - |  | Event B2-01 | 0.6 | >2.0 | - |
|  | | |  | Event B2-02 | >2.2 | - | - |
|  |  |  |  | Event B2-03 | >2.6 | - | - |
|  |  |  |  | Event B2-04 | 1.3 | >1.4 | - |
|  |  |  |  | $LR_{eff.}$ Event B2 | 1.4 | - | - |

^A^ Floc blanket clarification

^B^ Rapid sand filtration

^C^ Ballasted clarification

^D^ Ozone and granular activated carbon filtration

**Supplementary Table 2.** Raw and settled water turbidity levels in baseline and event conditions at drinking water treatment plants (DWTPs) A and B

|  |  | **Turbidity (NTU)** | | |
| --- | --- | --- | --- | --- |
|  | **Sample id.** | **Raw water** | **Settled water** | **Log-removal** |
| DWTP A | Event A1-01 | 6.14 | 0.65 | 1.0 |
|  | Event A1-02 | 7.80 | 0.65 | 1.1 |
|  | Event A1-03 | 8.37 | 0.66 | 1.1 |
|  | Event A2-01 | 13.5 | 0.72 | 1.3 |
|  | Event A2-02 | 15.8 | 0.78 | 1.3 |
|  | Event A2-03 | 27.1 | 0.78 | 1.5 |
| DWTP B | Baseline-01 | 6.99 | 0.66 | 1.0 |
|  | Baseline-02 | 6.51 | 0.74 | 1.0 |
|  | Event B2-01 | 14.74 | 0.97 | 1.2 |
|  | Event B2-02 | 12.12 | 0.82 | 1.2 |
|  | Event B2-03 | 16.42 | 0.80 | 1.3 |
|  | Event B2-04 | 15.09 | 0.69 | 1.3 |

| **▬** GLUC activity **▬ ▬** Turbidity **▬** Ammonia - NH_3_ ●● River flow rate | | | |
| --- | --- | --- | --- |
| DWTP A– Event A1 | | DWTP A– Event A2 | |
|  |  | |  |
| DWTP B– Event B1 | DWTP B– Event B2 | |  |
|  |  | |  |

**Supplementary Figure 1**. Short-term fluctuations in β-D-glucuronidase (GLUC) activity, turbidity, ammonia level, and river flow rate during event conditions at drinking water treatment plants (DWTPs) A and B.

| **Wastewater treatment plant - Influent** |  |
| --- | --- |
|  | |
| **Wastewater treatment plant - Effluent** |  |
|  | |

**Supplementary Figure 2**. Histograms for the concentrations of eight enteric viruses at the influent and effluent of a wastewater treatment plant located 5 kilometres upstream from drinking water treatment plant (DWTP) B

. Error bars represent the uncertainty in virus concentrations due to the analytical error and the random error in sample collection. Influent samples were collected on three occasions in 2018 on February 28, March 19, and March 26. Effluent samples were collected on February 28 and March 26. Orange glowing bars represent samples positive for ICC-qPCR

**R code for the quantification of virus concentrations**

#THE DATA.

copies =

vPCR =

vpellet=

vsample=

#------------------------------------------------------------------------------

# Package the data for shipping to JAGS:

dataList = list(

copies = copies ,

vPCR = vPCR ,

vpellet=vpellet,

vsample=vsample,

N = length(copies)

)

#------------------------------------------------------------------------------

# THE MODEL.

modelstring ="

model {

for( i in 1 : N ) {

#Likelihood

copies[i] ~ dpois(mu*vPCR[i])

rec[i] ~ dbeta( , )

conc=(mu*vpellet/vsample)/rec[i]

}

# Prior:

mu ~ dgamma(0.001, 0.001)

}

" # close quote for modelstring

writeLines(modelstring,con="model.txt")

#------------------------------------------------------------------------------

# RUN THE CHAINS

require(rjags)

parameters = c("conc")

adaptSteps = 2000 # Number of steps to "tune" the samplers.

burnInSteps = 1000 # Number of steps to "burn-in" the samplers.

nChains = 3 # Number of chains to run.

numSavedSteps=10000 # Total number of steps in chains to save.

thinSteps=1 # Number of steps to "thin" (1=keep every step).

nPerChain = ceiling( ( numSavedSteps * thinSteps ) / nChains ) # Steps per chain.

# Create, initialize, and adapt the model:

jagsModel = jags.model( "model.txt" , data=dataList ,

n.chains=nChains , n.adapt=adaptSteps )

# Burn-in:

cat( "Burning in the MCMC chain...\n" )

update( jagsModel , n.iter=burnInSteps )

# The saved MCMC chain:

cat( "Sampling final MCMC chain...\n" )

mcmcCoda = coda.samples( jagsModel, variable.names=parameters ,

n.iter=nPerChain , thin=thinSteps )

mcmcChain = as.matrix( mcmcCoda)

chainLength = NROW(mcmcChain)

# EXAMINE THE RESULTS

# Display diagnostics of chain, for specified parameters:

parameterNames = varnames(mcmcCoda) # get all parameter names

for ( parName in parameterNames ) {

diagMCMC( codaObject=mcmcCoda , parName=parName ,

saveName=fileNameRoot , saveType=graphFileType )

}

layout(matrix(1:6,nrow=2,byrow=TRUE))

postInfo = plotPost( mcmcChain[,"conc"] , xlab="conc" )
